# A Cost-Based Analysis of Anti-aging Products Across Four Major United States Retailers

**DOI:** 10.7759/cureus.46596

**Published:** 2023-10-06

**Authors:** Luis F Andrade, Loren E Hernandez, Kayla D Mashoudy, Maria J Lalama, Manya Saaraswat, Ryan J Scheinkman, Shasa Hu

**Affiliations:** 1 Dr. Phillip Frost Department of Dermatology and Cutaneous Surgery, University of Miami Miller School of Medicine, Miami, USA

**Keywords:** cost analysis, public health, disparities, photoaging, sunscreen

## Abstract

Background

In the field of aesthetic dermatology, there is currently very little data on affordability and cost analysis regarding cosmeceuticals as more demand from patients showing interest in cosmeceutical products to reduce and prevent aging continues to grow. Photoaging, a form of extrinsic aging from sun exposure, can be ameliorated by applying sunscreen and retinol products. Topical ascorbic acid and niacinamide have been shown to target the oxidative stress process that contributes to photoaging. These four products have been identified as the cosmeceutical ingredients with the most evidence-based data on photoaging prevention and treatment.

Objective

Given the demand for effective skin care, the paucity of data on cost differentiation, and the availability of cosmeceutical products, we analyzed the unit cost of four anti-aging products from major online and physical retailers in the United States. Such a cost comparison may facilitate more economically appropriate recommendations on skin care to consumers.

Methods and materials

We analyzed sunscreen, topical vitamin C (ascorbic acid), topical vitamin B3 (niacinamide), and topical vitamin A (retinol) products sold by four major United States retailers: Walmart, Ulta, Walgreens, and Amazon. The average cost in dollars per ounce (dollar/oz) was calculated for each product category at each retailer. Statistical analyses were done to determine statistical significance for each product category between retailers as well as between each category of product.

Results

Between the four retailers, Walmart offered the lowest cost per ounce for every product. In contrast, Amazon offered the highest cost per ounce for every product except for sunscreen. We also found that sunscreen products are less expensive per ounce as compared to retinol, ascorbic acid, and niacinamide products.

Conclusion

Dermatologists should be knowledgeable of product costs when providing patients with anti-aging product recommendations. Our study provides data on the financial cost by retail location of evidence-based anti-aging cosmeceuticals to better guide physicians in patient consulting and economical resource sharing.

## Introduction

Photoaging, a result of chronic sun exposure, presents itself clinically as dyspigmentation, loss of skin elasticity and turgor, and the development of telangiectasias and rhytides [1,2]. Ultraviolet light radiation (UVR) is the key culprit for photoaging, as it induces oxidative stress via the production of reactive oxygen species (ROS), further contributing to the aging process [3]. Approximately 80% of aging has been attributed to sun exposure. Therefore, photoprotection, mainly through the daily application of sunscreen, helps mitigate photoaging [4]. Currently, the American Academy of Dermatology (AAD) recommends that sunscreen should offer broad-spectrum protection against both ultraviolet A (UVA) and ultraviolet B (UVB), be water resistant for 40 to 80 minutes, and have a sun protection factor (SPF) of at least 30 [5]. Additionally, targeting oxidative stress via the topical application of antioxidants, such as ascorbic acid [6,7] and niacinamide [8-10], has been shown to abate photoaging of the skin. Furthermore, the application of topical retinoids, a derivative of vitamin A, has also been shown to ameliorate signs of photoaging [11].

The prevention of aging and the maintenance of a youthful appearance are highly sought after, as reflected by the $17-billion dollar global anti-aging industry [12]. Over the years, the demand from patients for anti-aging products has significantly increased. A study performed by market research company NPD Group in 2012 revealed that fewer than 20% of U.S. women between the ages of 18 and 24 considered the usage of anti-aging regimes to be important [13]. By 2018, another study by The Benchmarking Company found that the number had increased to more than 50% [14]. With the increase in demand, it is likely that patients will be more likely to ask their dermatology providers about anti-aging recommendations. Unfortunately, there are socioeconomic barriers for individuals to receive anti-aging care; these barriers include the varied cost and complexity of certain anti-aging regimens in addition to access to an informed dermatologist. According to the most recent US census [15], approximately 37.2 million people live in poverty. These differences in income can contribute to exacerbating healthcare disparities in the field of dermatology, particularly in the realm of photoaging. It is vital that dermatologists be mindful of their patients’ budgets when offering product recommendations to lessen their economic burden while also recommending products with the best level of evidence in photoaging benefits. Currently, sunscreen, topical vitamin C (ascorbic acid), topical vitamin B3 (niacinamide), and topical vitamin A (retinol) have the most evidence-proven results [16]. In this study, we aim to provide patients and dermatologists with a cost-based analysis of sunscreen, ascorbic acid, niacinamide, and retinol products sold at four major US retailers.

## Materials and methods

**Figure 1:**
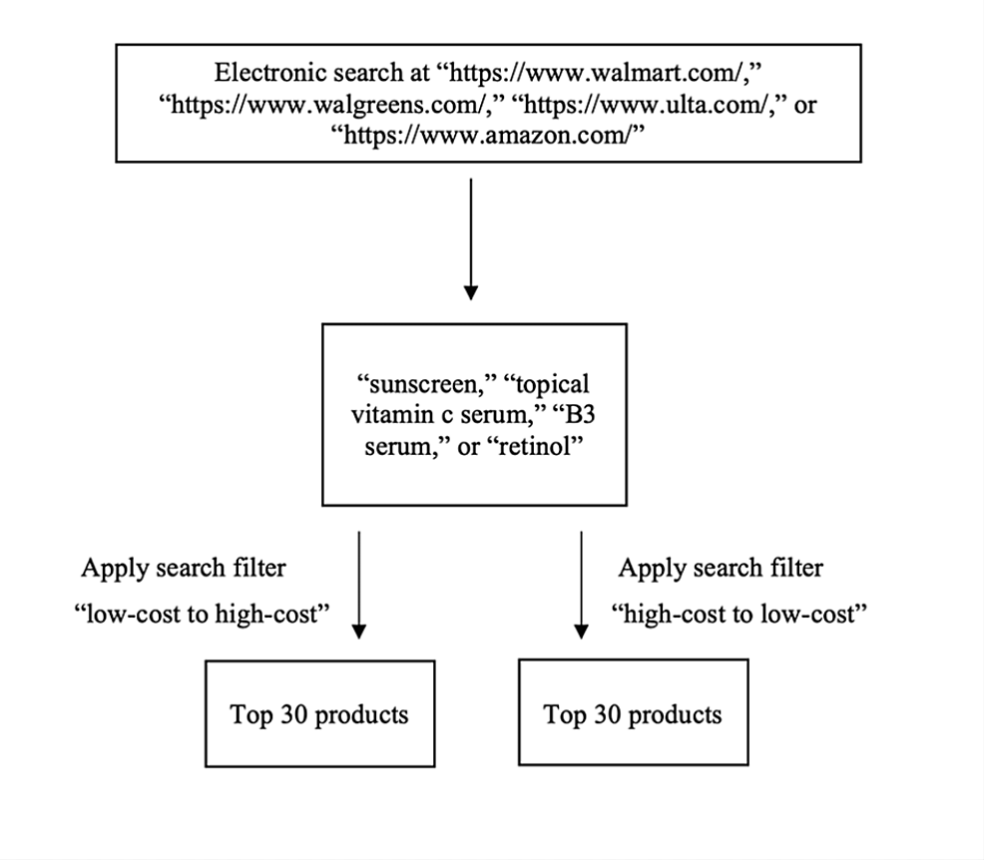
Flowchart diagram detailing the product search process

We analyzed sunscreen, ascorbic acid, niacinamide, and retinol products sold by four major United States (US) retailers: Walmart (Bentonville, AR), the second-largest retailer in the US; Walgreens (Deerfield, IL), the largest pharmacy in the US; Ulta (Bolingbrook, IL), a major US beauty retailer; and Amazon (Seattle, WA), the largest US retailer. These retailers were chosen as they constitute the largest US retailers that offer sunscreen, ascorbic acid, niacinamide, and retinol products to consumers according to the National Retail Federation 2022 list [17]. We queried “https://www.walmart.com/,” “https://www.walgreens.com/,” “https://www.ulta.com/,” and “https://www.amazon.com/” in April 2022. The following search terms were used to identify sunscreen, ascorbic acid, niacinamide, and retinol products, respectively: sunscreen, topical vitamin C, topical B3, and topical retinol. Products were filtered from low cost to high cost as well as from high cost to low cost, and the top 30 products from each search term were recorded as outlined in Figure 1. Four different members of the study team independently validated the search results to ensure that the same order of products resulted when each search filter was applied. This task was done for each of the retailer websites.

Results were filtered by cost to acquire a representative sample of the products offered at these four retailers. Products labeled or described as “lip balm,” “makeup,” or “moisturizer” were excluded. Sunscreen products lacking an SPF value on the label and products without a main packaging label were also excluded. Additionally, topical vitamin C, topical B3, and topical retinol products lacking labels that listed the percentage of the respective active ingredient were excluded. In cases where fewer than 30 products resulted after searching for the mentioned terms, all resulting products were analyzed. The average cost in dollars per ounce (dollar/oz) was calculated for each product category at each retailer. Calculations were adjusted based on the percentage of active ingredients listed on the products. The two-tailed student’s T-test was used to determine statistical significance for each product category between retailers. Analysis of variance (ANOVA) was used to determine statistical significance between each category of product. Statistical analyses were performed twice by two independent investigators to ensure the accuracy of the results.

## Results

A total of 796 products were identified and included in our analysis in Table 1. For sunscreen products, Walmart offered the lowest cost per ounce (2.0/oz) while Ulta offered the highest (22.6/oz). Out of the four retailers analyzed, Amazon offered the greatest cost per ounce for retinol, vitamin C, and vitamin B3 products at 76.2/oz, 125.9/oz, and 38.2/oz, respectively. However, these differences were insignificant for retinol products sold at Ulta (P = 0.06), vitamin B3 products sold at Ulta (P = 0.29), and Walgreens (P = 0.53). Walmart also offered the lowest cost per ounce for retinol, vitamin C, and vitamin B3 products at 19.8/oz, 17.7/oz, and 22.7/oz, respectively. These differences in average cost per ounce at the consumer level were statistically significant when comparing products between retailers; however, these differences were insignificant when comparing the average cost per ounce of vitamin B3 products sold at Ulta (P = 0.1). When comparing the average cost in dollars per ounce between products sold at all retailers in Table 2, sunscreen products were the least expensive per ounce (11.4/oz, P = 0.000025), followed by retinol (44.4/oz, P < .00001), vitamin B3 (59.1oz, P = 0.34), and vitamin C (59.4/oz, P = 0.000076). However, changes in cost per ounce were insignificant for vitamin B3 as compared to the other product types.

**Table 1:** Average cost US dollars per ounce of sunscreen, retinol, vitamin C, and vitamin B3 at four major US retailers ^*^Statistical significance determined as P < .05 with the two-tailed T-test

	Sunscreen (N, average cost in US dollars/ounce)	P-Value (Sunscreen)	Retinol (N, Average cost in US dollars/ounce)	P-Value (Retinol)	Vitamin C (N, average cost in US dollars/ounce)	P-Value (Vitamin C)	Vitamin B3 (N, average cost in US dollars/ounce)	P-Value (Vitamin B3)
Walmart	60, 2.02	Walmart vs Ulta P = .000046*; Walmart vs Amazon P = .006406*	60, 19.81	Walmart vs Ulta P < .00001*; Walmart vs Amazon P = 0.000101*	60, 17.7	Walmart vs Ulta P < .00001*; Walmart vs Amazon P = 0.001138*	34, 22.71	Walmart vs Ulta P = 0.176558; Walmart vs Amazon P = 0.030855*
Ulta	60, 22.64	Ulta vs Walgreens P = .015151*; Ulta vs Amazon P = .039698*	60, 48.64	Ulta vs Walgreens P = 0.026989*; Ulta vs Amazon P = 0.055996	60, 54.23	Ulta vs Walgreens P = 0.000134*; Ulta vs Amazon P = 0.031855*	22, 30.31	Ulta vs Walgreens P = 0.425005; Ulta vs Amazon P = 0.293034
Walgreens	60, 10.32	Walgreens vs Walmart P < .00001*	60, 35	Walgreens vs Walmart P = 0.000469*	26, 31.4	Walgreens vs Walmart P = 0.000206*	5, 34.18	Walgreens vs Walmart P = 0.009001*
Amazon	58, 10.54	Amazon vs Walgreens P = .947262	56, 76.24	Amazon vs Walgreens P = 0.003996*	57, 121.5	Amazon vs Walgreens P = 0.00436*	58, 38.22	Amazon vs Walgreens P = 0.534877

**Table 2:** Comparison of average cost US dollars per ounce between sunscreen, retinol, vitamin C, and vitamin B3 products ^*^Statistical significance determined as P < .05 with the analysis of variance (ANOVA) test

Product Type	N, Average cost US dollars/oz	P-Value
Sunscreen	238, 11.39	P = 0.000025*
Retinol	236, 44.39	P < .00001*
Vitamin C	203, 59.40	P = 0.000076*
Vitamin B3	119, 59.10	P = 0.309351

## Discussion

Our study finds significant differences in cost per ounce for a wide range of sunscreen, retinol, vitamin C, and vitamin B3 products analyzed between four major US retailers. We also found significant differences in cost per ounce between most product types, irrespective of retailer. Retailers that offer products with a greater average cost per ounce should consider adding less expensive products to their repertoire to better serve consumers with lower incomes. Having a lower socioeconomic status or living in a rural geographic location can be potential barriers to over-the-counter skin care products. Individuals in rural areas or even urban centers lacking a variety of physical retailers might be forced to utilize exclusively online-only retailers, which have the highest cost per ounce of all the retailers examined. One limitation of our study is that we didn’t include local, small retailers such as Navarro and Iras Discount Pharmacy. These smaller retailers may offer a more affordable variety of anti-aging products but can likewise be limited by location as opposed to more common chain retailers. For example, Navarro may have anti-aging products available at lower prices but may be located farther from an individual; that patient will now have to balance the time and travel costs with the monetary product costs to decide whether they will purchase their products from a large, online retailer or a smaller, physical retailer.

It has been shown that lower socioeconomic status and geographic location contribute to healthcare inequities for patients. Such patients have been shown to have less access to or use of dermatologists as well as poorer health outcomes [18]. Given that 37.2% of the US population lives in poverty, dermatologists need to be better equipped with cost-effective product options for their patients when asked for anti-aging product recommendations [19]. Since sunscreen products were the least expensive product as compared to retinol, vitamin C, and vitamin B3, patients who are budget-conscious and inquire about anti-aging product recommendations may be advised to focus on incorporating sunscreen as the most cost-effective addition to their skincare routine.

Moreover, our study included only 30 products for each search term and filtered results from low-cost to high-cost and vice versa, which may have limited our sample size. Further studies can expand upon this work by analyzing more products per search term and filtering or including all products without filter limitations. Nevertheless, our study provides an important benchmark analysis of the cost variability of common skin care products that address concerns about photoaging.

## Conclusions

Dermatologists should be knowledgeable of product costs when providing patients with anti-aging product recommendations. Our analysis of sunscreen, topical vitamin A, topical vitamin C, and topical vitamin B3 products sold at four major US retailers provides dermatologists with more information regarding the cost per ounce of product. A greater knowledge of the costs associated with products as well as better awareness of patient populations’ budgets given the varied economic landscape in the US helps ameliorate some of the economic burden placed on patients.
